# Role of store-operated Ca^2+^ entry and STIM2 in retinal neurodegeneration during glaucoma

**DOI:** 10.3389/fnagi.2025.1657590

**Published:** 2025-09-25

**Authors:** Sofiia Baranykova

**Affiliations:** Laboratory of Neurodegeneration, International Institute of Molecular and Cell Biology in Warsaw, Warsaw, Poland

**Keywords:** store-operated calcium entry (SOCE), STIM2, calcium homeostasis, microglia, retinal ganglion cells, retina, glaucoma

## Abstract

Ca^2+^ homeostasis is essential for glial cell activity and normal neuronal function, and store-operated Ca^2+^ entry (SOCE) is one mechanism that maintains it. The present review discusses the interplay between Ca^2+^ dysregulation and microglial activation in glaucomatous retinal degeneration. We examine the impact of Ca^2+^ homeostasis and SOCE on microglial function and their potential role in retinal ganglion cell degeneration and present the hypothesis that SOCE dysregulation may underlie glaucomatous pathology. This review suggests that targeting Ca^2+^ pathways in microglial cells can be a potential treatment for glaucoma.

## Introduction

Glaucoma is a pathology that is still not fully understood, has different subtypes, is often undiagnosed, and needs new treatment approaches. Some of its subtypes, such as retinal degeneration without intraocular pressure (IOP), need early diagnostic markers.

Glaucoma has a comprehensive mechanism that involves various cell types and pathways. The present review concentrates on Ca^2+^ signaling in microglial cells because of their involvement in inflammation and neurodegenerative processes (Heo et al., 2015; Michaelis et al., 2015). These observations are supported by our data. The information that is presented in this mini-review indicates that proteins that are involved in Ca^2+^ homeostasis in microglial cells may be used as diagnostic markers and may be potential targets for therapeutic strategies for glaucoma.

## Definition and types of glaucoma

Glaucoma comprises a group of diseases that affect approximately 100 million people worldwide, often leading to vision loss. Its major features are optic nerve degeneration and the loss or dysfunction of retinal ganglion cells (RGCs) (Crish and Calkins, 2011; Zhang et al., 2021). In addition to these factors, mitochondrial dysfunction and neuroinflammation are also observed (Saccà and Izzotti, 2014).

Glaucoma can be classified based on age (congenital, juvenile or adult), etiology (primary, with no identifiable cause or secondary, resulting from previous ocular trauma), and the location of obstruction in the drainage system of the eye (primary open angle or primary angle closure glaucoma) (Pang and Clark, 2020; Ekici and Moghimi, 2023).

In most cases, glaucoma is primarily associated with elevated intraocular pressure (IOP), which results from dysfunction in outflow pathways of aqueous humor from the eye (Jayaram et al., 2023). However, it can also occur without an elevation of IOP, which is referred to as normal-tension glaucoma or low-tension glaucoma and can be caused by vascular dysregulation, impairments in blood flow, glymphatic system failure, or other factors (Shinozaki et al., 2024).

The majority of all these types of glaucoma have common players, such as microglial cells (Duarte, 2021; Wang et al., 2014; Somerville et al., 2025), mostly because of their involvement in neuroinflammation and retinal ganglion cell degeneration. The present review discusses both findings from the literature and our own data, emphasizing that microglial activity during retinal neurodegeneration may be significantly influenced by Ca^2+^ homeostasis, particularly the store-operated Ca^2+^ entry (SOCE) pathway. Additionally, zebrafish with *stim2* gene knockout may be a promising animal model of glaucomatous pathology.

## Pathophysiology and models of glaucoma

Environmental factors, such as air pollution and an inappropriate diet (Almarzouki, 2024), may be risk factors for glaucoma development. However, there is no clinically proven evidence that cigarette smoking, alcohol consumption, or excessive caffeine intake can cause this disease (Stuart et al., 2023). The most crucial factor could be an imbalance in the consumption of essential fatty acids or frequent salt intake. Glaucoma has been shown to be strongly associated with chronic stress and anxiety (Stuart et al., 2023). However, many different factors contribute to the development of glaucoma (Stuart et al., 2023).

To gain a complete picture of glaucoma's pathogenesis, researchers must employ various animal models that reflect its multifaceted origins. Most models are based on IOP pathology and are called ocular hypertension models (Reinehr et al., 2024). They are induced by blocking drainage pathways, injecting hypertonic saline, or introducing mutations that block aqueous humor outflow. Proinflammatory pathways during glaucoma can be activated by high IOP, along with other factors (e.g., oxidative stress), which in turn lead to the release of signaling molecules by glial cells and RGCs that contribute to cellular damage and death (Bugara et al., 2024).

Hyperactivation of the 2′3′-cyclic guanosine monophosphate–adenosine monophosphate–stimulator of interferon gene (cGAS–STING) signaling pathway in microglial cells is responsible for RGC loss and vision deterioration (Liu et al., 2024). cGAS–STING signaling proteins are located in the endoplasmic reticulum and Golgi apparatus. In a glaucoma model in mice, 95% of STING was shown to colocalize with microglia markers (Liu et al., 2024). However, microglial activation does not depend on this signaling. cGAS–STING signaling is responsible for activation of the innate immune response by sensing double-stranded DNA (dsDNA) in the cytoplasm. Such dsDNA is observed in RGCs during their degeneration. When cGAS–STING is activated, it induces the expression of cytokines and chemokines by microglia and other innate immune cells. The ablation of this pathway has been shown to prevent the apoptosis of RGCs and visual deterioration (Zhang et al., 2024; Wu et al., 2023). Additionally, cGAS–STING signaling has been proposed to be highly connected to cellular stress (Schmid et al., 2024).

Our recent ultrastructural analyses revealed mitochondrial damage and cristae loss in photoreceptors in *stim2* knockout zebrafish larvae (Baranykova et al., 2024), which are hallmarks of cellular stress. Considering the connection between STING signaling, mitochondrial stress, and glaucomatous neurodegeneration, these data support a possible link between retinal neurodegeneration and Ca^2+^ dysregulation. Overall, our evidence suggests that SOCE dysfunction, particularly attributable to the loss of stromal interaction molecule 2 (STIM2) protein, may play an important role in the pathogenesis of glaucoma.

Other genetic models of IOP-dependent glaucoma include mutations of the *Prss56* gene in E50Ktg mice, which mimics the angle closure subtype (i.e., normotensive glaucoma with RGC loss) (Amato, 2022). The most widely used and common models are DBA/2J mice and myocilin mutant mice, which carry a point mutation in Tyr423His in the *Prss56* gene and exhibit an increase in IOP and age-dependent glaucoma progression (Amato, 2022). Studies of age-dependent eye diseases show a significant increase in the pathogenic activity of microglia with age, which in turn can cause chronic inflammation (Ahmad and Subramani, 2022).

Models to study IOP-independent cases usually involve excitotoxicity, immune modulation, ischemia or reperfusion, optic nerve crush, and gene modification (Tsai et al., 2024). In some IOP-independent rat models, the peak of the microglial response appears at initial stages (i.e., day 3 in the case of *N*-methyl-D-aspartate-induced retinal degeneration) (Kuehn et al., 2017). The activity of microglial cells decreases thereafter (Kuehn et al., 2017).

None of the existing models provide a complete picture of glaucoma. Multiple model systems are needed to obtain a more comprehensive picture of the disease (Bugara et al., 2024). Most available treatments to improve vision in glaucoma are designed to decrease IOP, but there is a need to focus on possible neuroprotective pathways in IOP-independent cases (Bugara et al., 2024).

Given the multifactorial nature of glaucoma and limitations of current IOP-based models, there is an increasing need to investigate IOP-independent mechanisms, especially those that are related to neuroinflammation and cellular stress pathways. Among these, Ca^2+^ homeostasis and its regulators appear to be critical factors that influence RGC survival and microglial activation. Thus, SOCE and its key components (i.e., STIM1, STIM2, and Ca^2+^ release-activated Ca^2+^ channel protein [ORAI]) deserve further investigation. The next section discusses the role of STIM2-dependent SOCE in neurodegenerative processes, highlighting its potential contribution to glaucomatous retinal damage.

## STIM2-dependent SOCE: role of STIM2 in neurodegenerative mechanisms

SOCE is potentially an important mechanism for Ca^2+^ regulation in all metazoan cells (Prakriya and Lewis, 2015; Korshunov and Prakriya, 2025). It is a major Ca^2+^ influx pathway in non-excitable cells (Wegierski and Kuznicki, 2018). The main components of SOCE are STIM1 and STIM2 (which are located in the endoplasmic reticulum and trigger Ca^2+^ influx when endoplasmic reticulum Ca^2+^ levels drop) and ORAI1, ORAI2, and ORAI3 (which are located in the plasma membrane and enable Ca^2+^ ions to enter). Additional components include transient receptor potential canonical channels (TRPCs; which modulate SOCE), SARAF (store-operated Ca^2+^ entry-associated regulatory factor which prevents Ca^2+^ overload), and Ca^2+^ release-activated channel regulator 2A (CRACR2A; a modulator of immune cells) (Srikanth et al., 2010; Wegierski and Kuznicki, 2018; Prakriya and Lewis, 2015). When Ca^2+^ in the endoplasmic reticulum is sufficient, STIM remains inactive. After Ca^2+^ levels decrease to a particular level, STIM changes its conformation, oligomerizes, migrates to endoplasmic reticulum—plasma membrane junctions, and binds to ORAI, which allows Ca^2+^ to enter the cytoplasm.

Previous studies (Baranykova et al., 2024; Wasilewska et al., 2023) have shown that *stim2* knockout in *Danio rerio* leads to a glaucoma-like phenotype. Mutant zebrafish exhibit signs of activated microglia (i.e., an increase in the *anxa3a* gene), a loss of RGCs and their dendrite numbers, a decrease in γ-aminobutyric acid-ergic cells and photoreceptors, downregulation of the *ins* gene, and mitochondrial disruptions. These findings strongly suggest that STIM2 is essential for retinal homeostasis and microglial function. A previous study (Wasilewska et al., 2023) showed that *stim2*-deficient zebrafish exhibit impairments in visual behavior and greater sensitivity to hypoxia, which supports the hypothesis that STIM2 might regulate neuroprotective mechanisms in the retina.

**Figure 1:**
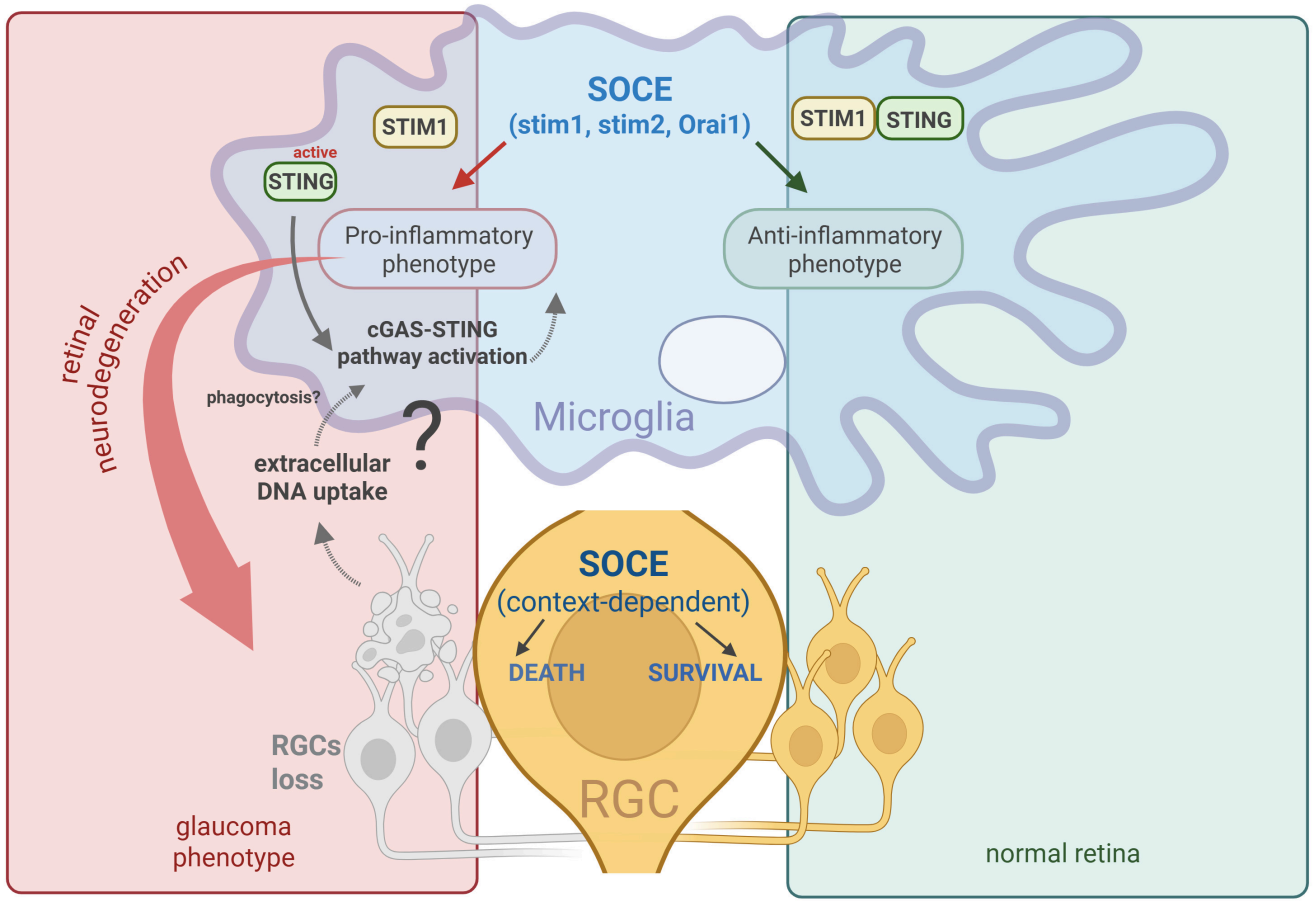
Schematic illustration of the possible role of SOCE in microglial cells and the progression of RGC loss during glaucomatous neurodegeneration.

Recent evidence has shown a link between SOCE and STING activation in neurons. Woo et al. (2024) demonstrated that STIM1 directly interacts with STING, in which it helps keep STING in the endoplasmic reticulum when the cell is in a resting state. During inflammation, STIM1 detaches from STING, followed by STING activation and neurotoxic signaling. Interestingly, SOCE activation serves as a trigger for this process. In neurons that lack STIM1 protein, STING activation increases. These findings suggest that the dysregulation of STIM1 or STIM2 may make neurons more sensitive to STING-related stress, which can explain how Ca^2+^ imbalance and nerve cell loss occur in such diseases as glaucoma (Figure 1).

Based on prior data (Baranykova et al., 2024; Wasilewska et al., 2023), the disruption of STIM2 is hypothesized to cause microglial cell activation, which in turn leads to RGC loss (Figure 1). Still not fully understood is how SOCE, particularly STIM2, is involved in glaucomatous neurodegeneration. Nevertheless, our recent studies provide some new evidence of a link between SOCE dysregulation and RGC loss in the retina (Baranykova et al., 2024; Wasilewska et al., 2023).

## Ca^2+^ homeostasis in retinal ganglion cells

The survival of RGCs during axon injury depends on baseline Ca^2+^ levels. Cells with lower baseline Ca^2+^ levels degenerate more frequently during neuropathology than those with higher levels (McCracken et al., 2023). This phenomenon means that Ca^2+^ homeostasis determines the survival and resilience of RGCs. SOCE participates in the regulation of baseline Ca^2+^ levels; thus, a reasonable assumption is that its components regulate RGC survival during neuropathology (McCracken et al., 2023; Molnar et al., 2012). During glaucomatous neurodegeneration, Ca^2+^ homeostasis is significantly altered in RGCs, particularly light-driven Ca^2+^ dynamics (Quintero et al., 2022). These data support a regulatory role for Ca^2+^ in RGCs. However, unclear is how Ca^2+^ signals influence RGC death and survival (McCracken et al., 2023).

One suggestion is that Ca^2+^ may be involved in IOP-dependent and -independent glaucoma (Kastner et al., 2023). The involvement of Ca^2+^ in IOP-dependent diseases may occur through mitogen-activated protein kinase/extracellular signal-regulated kinase and nuclear factor-κB pathways (Wang et al., 2023). The TRPC5 Ca^2+^ channel, which regulates Ca^2+^ levels, is a negative regulator of RGC axonal outgrowth (Oda et al., 2020).

In summary, correct Ca^2+^ regulation is essential for the survival and function of RGCs, especially under stress conditions during glaucoma. Disruptions of Ca^2+^ signaling, particularly through SOCE, appear to be involved in RGC degeneration. However, the exact mechanisms by which Ca^2+^ imbalance causes RGC death (i.e., either directly or through interactions with other retinal cell types like microglia) remain to be explored. Further studies of these interconnected pathways are necessary to develop more advanced treatment strategies for glaucoma.

## Ca^2+^ homeostasis in microglial activation

Several studies have shown that SOCE might influence the activity of microglial cells (Heo et al., 2015; Wegierski and Kuznicki, 2018). Changes in expression of the SOCE channels Orai1 and TRPC1 have been shown to regulate the transformation of microglia toward either a proinflammatory or antiinflammatory state (Nascimento Da Conceicao et al., 2021). For example, the antiinflammatory phenotype can be achieved through Orai1 activation. Because Orai1 is functionally connected to STIM2, any changes in STIM2 levels or its structure can be expected to alter microglial function.

Mechanistically, SOCE regulates microglial functions primarily through Ca^2+^-dependent phagocytosis and motility. STIM1 and STIM2 sense endoplasmic reticulum Ca^2+^ depletion and activate Orai1 channels, generating Ca^2+^ influx that sustains essential microglial processes. Blocking or knocking down STIM1, STIM2, or Orai1 impairs uridine diphosphate-induced phagocytosis and FcγR-mediated phagocytosis, demonstrating the dependence of microglial clearance functions on SOCE (Michaelis et al., 2015; Heo et al., 2015; Sogkas et al., 2015). STIM1 also facilitates Ca^2+^-dependent phagosome–endoplasmic reticulum interactions, and its ablation decreases phagocytic efficiency (Nunes et al., 2012). The disruption of STIM2 has been shown to reduce inflammation and apoptosis, indicating a broader role in regulating Ca^2+^-dependent cellular responses (Ye et al., 2024). Additionally, acute microglial motility is Ca^2+^-dependent and requires STIM1 (Lim et al., 2017). These findings indicate that proper SOCE activity is essential for microglial surveillance and debris clearance (Hallett, 2020).

Disruption of the STIM/Orai system can have downstream consequences for neuronal survival. For example, in zebrafish, *stim2a* and *stim2b* double knockouts exhibit RGC loss that resembles glaucoma (Baranykova et al., 2024). This suggests that Ca^2+^ dysregulation may contribute to both microglial dysfunction and neuronal damage.

Both RGC resilience and microglial function are interconnected with, and depend on, Ca^2+^ levels. Different scenarios can be assumed: (i) microglial activation or other functional changes that are attributable to Ca^2+^ pathways promote RGC damage, (ii) RGC loss can appear independently of microglial activation through Ca^2+^ dysregulation in RGCs, or (iii) RGC degradation (through Ca^2+^ dysregulation) triggers microglial activation. It is difficult to establish which event occurs first and which is secondary. It is supposed that they are interdependent, in which Ca^2+^ dysregulation plays a significant role.

## Dual role of microglia in glaucoma: beneficial or detrimental?

Microglia perform various functions, including immune surveillance, synaptic refinement, neurotrophic support, and debris clearance. During neurodegenerative disorders, microglia are activated and increase the secretion of cytokines and phagocytic activity. Under normal conditions, the retina contains 0.3–1% microglial cells, which are located mostly in the inner plexiform layer or outer plexiform layer. In glaucoma, microglial cells undergo significant changes in morphology, cytokine synthesis, number, and distribution (Noailles et al., 2014). Activated microglia have been detected in retinitis pigmentosa, age-related macular degeneration, diabetic retinopathy, uveitis, and glaucoma. In all of these diseases, there is a loss of photoreceptor cells and/or RGCs (Wang and Cepko, 2022; Ramirez et al., 2017; Wang et al., 2021).

During retinal degeneration, an increase in the number of immunoglobulin G deposits and an increase in microglial activity have been detected. Microglial cells were observed near the site of immunoglobulin G deposition, and their increase was observed in the RGC layer and inner plexiform layer. The mechanism that leads to RGC loss appears to be connected to microglia activation and inflammatory processes (Joachim et al., 2012).

Microglia are highly interconnected with other glial cells (e.g., Müller cells) (Carpi-Santos et al., 2022). Hu et al. (2021) showed that experimental glaucoma is observed during the activation of microglia and Müller cells. Müller cell activation occurs through the adenosine triphosphate/P2X7 receptor pathway (Xu et al., 2022). Inflammation during glaucoma is suggested to be intensified by an interplay between microglia and Müller cells.

The role of microglia in glaucoma is not fully understood; it can be either detrimental or ameliorative. The depletion of microglia decreases cell survival (Ishikawa et al., 2023; Diemler et al., 2024) and worsens the process of regeneration or aggravates neurodegeneration (Diemler et al., 2024). Other studies reported that the depletion of microglia had no significant effect on visual function during glaucoma (Tan et al., 2021) or was a promising approach for neuroprotection (Ishikawa et al., 2023). Microglia fix visual circuits by engulfing synaptic elements during development (Schafer et al., 2012), interacting with dendritic spines in an activity-dependent manner (Tremblay et al., 2010) and mediating ocular dominance plasticity (Sipe et al., 2016), with roles in adult synapse remodeling (Wang et al., 2016). In glaucoma, these functions may deteriorate, potentially promoting synapse and RGC loss.

One hypothetical model of events in the retina during glaucoma was proposed to explain these inconsistencies (Ramirez et al., 2017). According to this model, microglial activation in the outer plexiform layer, inner plexiform layer, ganglion cell layer, and nerve fiber layer during initial stages of the disease plays mostly a protective role. However, in the case of chronic disease, activated microglial cells are mostly detrimental, and their blockade can reduce pathological progression (Ramirez et al., 2017).

These observations indicate that whether microglia are detrimental or beneficial depends on such factors as disease stage and the presence of mutations or dysregulated cell pathways (Ahmad and Subramani, 2022). Therefore, radical therapeutic approaches, such as microglia depletion, blockade, or activation, cannot be considered. Treatments should focus on correcting intracellular microglial pathways, such as Ca^2+^ homeostasis, because of its significant regulatory role in these cells. For example, blocking a change in the proinflammatory phenotype to the antiinflammatory phenotype can be achieved by modifying Ca^2+^ channel activity (Nascimento Da Conceicao et al., 2021).

## Therapeutic implications

The immune system plays a crucial role in the pathogenesis of glaucoma. Much data point to autoimmune aspects of this disease. However, the role of microglial cells in glaucoma is far from being fully understood (Tsai et al., 2024). Some glaucoma patients have specific antibodies against retinal antigens. Because some retinal diseases exhibit microglial activation, some treatments are designed to target microglia (Wang and Cepko, 2022). There are different strategies to target microglia in the eye, such as depleting microglia with specific chemicals or radiation, reprogramming microglia, and blocking cytokine activity or phagocytosis (Wang and Cepko, 2022). The behavior of microglial cell populations varies in various locations of the eye (Wang and Cepko, 2022). One way to deplete microglial cells is to use pexidartinib. This chemical inhibits colony stimulating factor 1 receptors that are expressed in microglia. However, several experiments have reported no benefits of such depletion (Wang et al., 2019).

Recently, Zhou et al. (2024) performed experiments using a mouse chronic ocular hypertension model to understand how non-coding RNA molecules that play role in regulating inflammation can influence glaucoma. Downregulation of the interleukin-1 receptor-associated kinase 1/tumor necrosis factor receptor-associated factor 6/nuclear factor-κB signaling pathway in microglia after transfection with miR-146a-5p reduced neuroinflammation and increased the survival of RGCs, suggesting that microglia may be a viable therapeutic target for glaucoma (Joachim et al., 2012).

## Summary

The loss of RGCs in glaucoma can have multiple causes. As described above, microglial cell dysfunction and Ca^2+^ homeostasis dysregulation can be linked to this disease. Microglia participate in glaucoma development via inflammation, phagocytosis, and other processes. In RGCs, proper Ca^2+^ levels determine survival, whereas in microglia, Ca^2+^ levels dictate cellular activity and whether it exhibits an anti- or proinflammatory phenotype. Changes in Ca^2+^ levels influence both microglial cells and RGCs, thereby complicating the identification of which processes initiate the pathology. For example, can activated microglia change Ca^2+^ homeostasis in RGCs to induce their neurodegeneration? It is suggested that proteins involved in Ca^2+^ homeostasis in microglial cells could be targets for anti-degeneration treatment.
